# Genes, guts, and microbes: decoding host-driven microbial regulation using intestine-specific conditional knockouts

**DOI:** 10.3389/fimmu.2025.1674913

**Published:** 2025-10-30

**Authors:** Priyankar Dey

**Affiliations:** Department of Biotechnology, Thapar Institute of Engineering and Technology, Patiala, Punjab, India

**Keywords:** intestine, gut, knockout, microbiome, host-microbe interaction

## Abstract

This narrative review underscores the influence of host genetics in actively regulating gut microbiota composition and function, highlighting the distinctive advantages of intestine-specific conditional knockout (cKO) models in gut microbiome research. In contrast to whole-body knockouts or germ-free animals, these precision models, enabled by Cre-loxP technology, eliminate confounding systemic effects to elucidate how localized host genes within intestinal cells regulate the gut microbial ecology. The review identifies three fundamental host-driven regulatory mechanisms through the analysis of specific gene deletions: (1) barrier integrity (e.g., mucus and junction proteins), (2) immune defenses (e.g., antimicrobial peptides and glycan synthesis), and (3) metabolic signaling (e.g., bile acid receptors and glucose transporter). These pathways jointly impose microbial symbiosis, and their disruption leads to dysbiosis characterized by increased abundance of pathobionts (e.g., *Escherichia, Proteobacteria*), directly connecting host genetics to inflammatory and metabolic disorders. This host-centric viewpoint emphasizes the gut as an active regulator, rather than a passive microenvironment for the microbiota, providing significant insights for creating tailored therapeutics that focus on host pathways to restore microbial balance in disorders such as inflammatory bowel diseases.

## 1 Introduction

The complex interplay between the host and gut microbiota is essential for the overall health and disease. The impact of microbiota on host physiology is well-documented (1–3); however, comprehending the reciprocal influence of the host on the gut microbial abundance and diversity is a vital field of research. Historically, research has predominantly concentrated on the effects of microbial communities on the host, resulting in a notable deficiency in comprehending the active mechanisms by which host systems, especially the intestinal components, affect their microbial residents (4–6). Germ-free (GF) animals, despite widely acknowledged physiological defects (7, 8), have played a crucial role in understanding the impact of gut microbes on the host health. These include understanding the crucial role of gut microbiota in immune maturation and functionality, energy metabolism, metabolic disease, and the developmental process (9, 10).

Similarly, knockout mice, genetically modified to be deficient in host-specific genes, plays an essential role in elucidating causal relationships among host genetics, the gut microbiota, and human health (**Table 1**). Essential roles include the immune genes (e.g., TLRs, NOD2, IL-10) in regulating microbial populations and maintaining barrier integrity, hence establishing a clear connection between dysbiosis and inflammatory disorders such as inflammatory bowel diseases (IBD) (30). Knockouts of metabolic signaling genes demonstrate that host genes regulate microbiome-mediated processes such as bile acid metabolism and dietary energy harvesting, influencing obesity and diabetes (31). Moreover, knockouts delineate host receptors crucial for detecting microbial metabolites like short-chain fatty acids (SCFA) and indoles, influencing inflammation and epithelial integrity (3). These models replicate human disease susceptibilities. affirming microbiome dependency and uncovering treatment targets, thereby clarifying the essential host mechanisms via which the microbiome significantly influences health. Nevertheless, intestine-specific conditional knockouts (cKO) provide a significant advantage over whole-body knockouts in the investigation of host-microbiota interactions. cKO animal models can be created using a wide variety of techniques such as Cre-loxP recombination system, inducible Cre-loxP systems, Flp-FRT recombination system, Dre-rox recombination system and CRISPR-Cas9 for tissue-specific knockout, that provide a robust method for facilitating tissue- or cell-specific gene deletion (32). The Cre-loxP system is generally used for the creation of mice with intestinal cKO (33), which entails mating a mouse harboring a target gene flanked by loxP sites with a mouse that expresses the Cre recombinase enzyme regulated by an intestine-specific promoter (34). In the resultant double-transgenic progeny, Cre selectively excises the floxed gene in intestinal cells, facilitating precise, tissue-specific gene deletion. Inducible systems such as CreERT2 are employed for temporal control, wherein Cre activity relies on tamoxifen administration, facilitating gene deletion at a designated moment throughout adulthood (35). Thus, this accuracy is essential for defining the specific, localized influence of host genes on the gut microbiota without interference from systemic effects. The selective disruption of genes in the intestinal epithelium or immune cells, circumvents the confounding systemic developmental anomalies, metabolic disorders, or premature mortality often seen with whole-body knockouts. This precision delineates the localized impacts of host genes on microbial colonization, community composition, metabolite detection, and barrier functionality within the intestinal milieu, enabling direct exploration of gut-specific processes.

**Table 1:** Summaries of studies showing the impact of whole-body knockout on the gut microbiota. .

Knocked-out gene	Notable microbiota impact	Health/Disease outcome	Reference
Innate Immune Sensing & Signaling (Pattern Recognition Receptors & Pathways)			
TLR4	↑Firmicutes: Bacteroidetes, Proteobacteria	Resistant from diet-induced obesity and systemic inflammation	(11)
TLR5 (Toll-like receptor 5)	Altered microbiota; metabolic syndrome phenotype transmissible through microbiota	Metabolic syndrome (weight gain, insulin resistance, dyslipidemia)	(12)
NLRP6 (NOD-like receptor P6)	↑ *Prevotellaceae* (Bacteroidetes) and TM7 phyla	Spontaneous colonic inflammation; aggravated DSS colitis	(13)
NLRP3 (NOD-like receptor P3	↑ *Prevotellaceae*	Aggravated effects of DSS colitis	(13)
Dectin-1/2 (Clec7a/Clec4n)	↑ *Blautia* and other Lachnospiraceae in Dectin-1/2 double KO mice	Strongly reduced DSS colitis severity (protection driven by microbiota)	(14)
MyD88 (Myeloid differentiation primary response 88)	↑ Proteobacteria in MyD88-suppressed colitis model	Unresolved/increased colitis severity; heightened inflammasome (NLR) signaling	(15)
CARD9 (Caspase recruitment domain 9)	Altered fungal–bacterial ecology (gut dysbiosis) upon CARD9 deletion	Increased susceptibility to colitis and colitis-associated cancer	(16)
Adaptive Immune System			
Rag1 (Recombination Activating Gene 1)	↓ Probiotic Lactobacillales; Enterobacteriales completely depleted; ↑ *Akkermansia muciniphila* in the colon.	Severe dysfunction of adaptive immunity; Microbial immune evasion	(17)
p53 and β chain of the T-cell receptor (TCR)	Not studies	Spontaneous colorectal tumor	(18)
Immunoglobulin A (IgA)	↑ Anaerobes and spore-forming Gram-positive bacteria (SFB) in the proximal intestine.	Restoring IgA levels inhibits SFB proliferation and restored the gut microbial community diversity.	(19)
Gut Barrier & Mucosal Immunity			
Muc2 (Mucin 2)	↑ *Akkermansia muciniphila*	Chronic colitis associated with modified brain glycine metabolism and behavior	(20)
ACE2 (Angiotensin-converting enzyme 2)	↑ Deferribacteres, Parasutterella; ↓ SCFA-producing genera (e.g. *Marvinbryantia, Alistipes*)	Impaired energy metabolism; insulin resistance, dyslipidemia	(21)
IL-10 (Interleukin-10)	↓ Microbial diversity; ↑ Proteobacteria and *Escherichia coli*	Spontaneous colitis (IBD model)	(22)
Metabolism, Neurotransmission & Gut-Brain Axis Signaling			
TGR5 (Takeda G-protein receptor 5)	↓ Microbial diversity; ↑ *Anaeroplasma*, *Prevotella*, *Staphylococcus*, *Jeotgalicoccus*, *Helicobacter*; ↓ *Bifidobacterium*	Anxiety- and depression-like behaviors (↓5-HT levels in brain/serum)	(23)
PYY (Peptide YY)	HFD-induced shift to ↑ Bacteroidetes (esp. *Alistipes, Parabacteroides, Muribaculum*) in PYY^-/-	Altered intestinal barrier (↑ CLDN2 tight-junction expression); no overt inflammation	(24)
5-HTT (Serotonin transporter, *Slc6a4*)	↓ *Allobaculum*, *Bifidobacterium*; ↑ *Dorea*, *Prevotella* in 5-HTT^-/- mice	Anxiety- and depression-like behaviors (altered gut–brain axis)	(25)
Leptin	↑Firmicutes: Bacteroidetes.	High food intake; Obese mice; Increased microbial energy harvesting; High monosaccharide and short-chain fatty acids (SCFAs)	(26)
TAAR9 (Trace amine-associated receptor 9)	Altered microbiome; ↑*Saccharimonadaceae*	↓ LDL cholesterol levels (improved lipid profile)	(27)
Sigma-1 receptor (Sig-1R)	↓ *Alistipes*, *Alloprevotella*, and *Lleibacterium*; Dysbiosis reproducible via FMT.	Depression-like behaviors (less immobility time, lethargy); Depression reversed by antibiotic and reproduced by FMT; Supressed cAMP/CREB/BDNF signaling.	(28)
ApoE (Apolipoprotein E)	Gut microbiota (vs germ-free) produces protective metabolites (e.g. equol, indoles) that attenuate plaque	Atherosclerosis (gut microbiota presence reduces arterial plaque formation)	(29)

The intestinal epithelium functions as a vital interface, establishing a dynamic barrier that separates the luminal environment from the underlying host tissues (36). This singular layer of specialized epithelial cells, comprising Paneth cells, goblet cells, tuft cells, enteroendocrine cells, and absorptive enterocytes, executes many roles crucial for preserving gut barrier integrity, immune surveillance, and nutrient processing (37). This characterizes the intestinal epithelium not only as a passive barrier but as an active, multi-functional entity that directly influences the microbial environment through several cell-specific methods. Disruptions in this fragile equilibrium can result in dysbiosis and other gastrointestinal and systemic disorders (38). Thus, this review exclusively summarizes the findings derived from intestine-specific cKO studies that have shown a direct influence on the gut microbiota, elucidating the active participation of the host in constructing its microbial environment.

## 2 Intestine-specific conditional knockouts and their influence on gut microbiota

Intestine-specific cKO models have yielded critical insights into the direct influence of the host on the composition of gut microbial populations. By selectively suppressing genes in the intestinal compartments, researchers can identify specific host factors and their mechanisms of action on the microbiota. The subsequent sections classify these findings according to the principal host function under examination.

### 2.1 Modulating intestinal barrier integrity and mucus production

**Figure 1:**
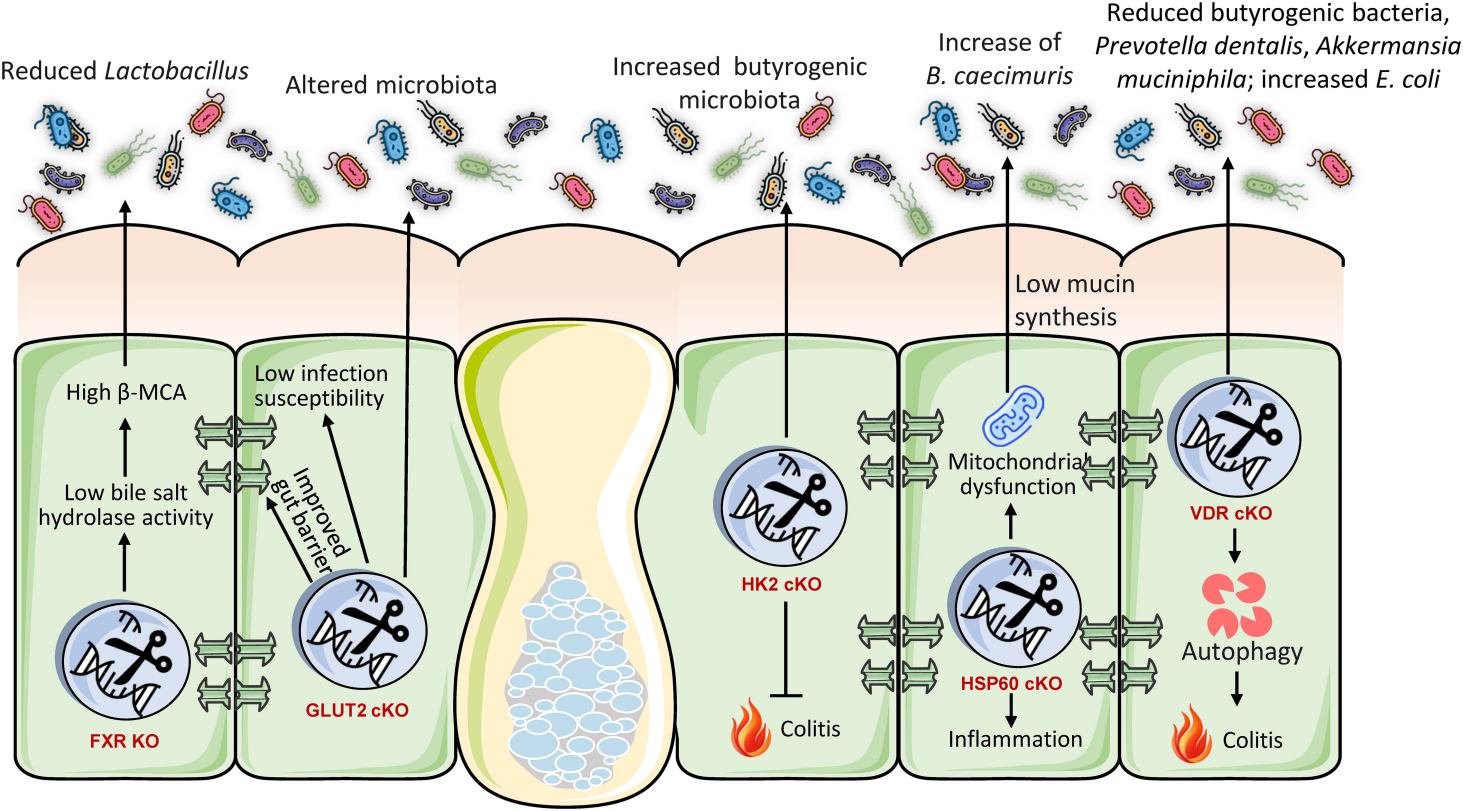
Intestine-specific knockout models illustrate that host genes are essential for preserving gut barrier integrity and a healthy microbiome. Deficiencies in genes that regulate mucus (SPP1), tight junctions (CLDN7), the cytoskeleton (ACF7), miRNAs (DICER1), or ion transport (NHE3) undermine the barrier and induce dysbiosis. This consistently leads to a reduction of beneficial symbionts, such as butyrate-producing Firmicutes, and an increase in pro-inflammatory pathobionts like Proteobacteria and *Escherichia*. These findings collectively demonstrate that host genes actively establish a selective environment to avert dysbiosis, with barrier failure serving as a prevalent mechanism that facilitates disease-associated microbial alterations.

The intestinal barrier, consisting of a mucus layer, epithelial cells, and tight junctions, serves as the principal interface between the host and its luminal microorganisms (3). cKO studies have identified various host genes essential for preserving this barrier and, as a result, influencing the gut microbiota (**Figure 1**). For instance, the *Spp1* (secreted phosphoprotein 1) gene encodes osteopontin (OPN), a highly phosphorylated and glycosylated acidic secreted protein that plays essential functions in immunological modulation, inflammatory responses, and cell adhesion (39). The intestine-specific conditional deletion of *Spp1* in mice substantially altered the expression of genes associated with immune control and lipid metabolism in the intestinal transcriptome (39). Metagenomic study of these animals indicated significant alterations in gut microbial diversity and anticipated metabolic pathways related to digestion, absorption, and fat metabolism (39). These findings indicate that *Spp1* is crucial for sustaining gut microbial balance and for modulating host lipid metabolism and immunological responses (39). Subsequent analysis revealed that intestine-specific *Spp1* cKO animals displayed a significantly lowered colonic mucus layer and reduced mucin staining intensity, signifying a deficiency in both the quantity and functionality of the goblet cells (39). The depletion of the mucus layer likely undermines the intestinal barrier, thereby heightening vulnerability to infections and inflammation (39). Thus, the complex involvement of *Spp1* in gut homeostasis is evident i.e., it serves not only as a general immune modulator but also as a vital element affecting the physical barrier via mucus production and the metabolic environment, both of which directly influence bacteria colonization and function. The interaction among immunological, metabolic, and physical barrier activities facilitated by a singular host protein such as *Spp1* highlights the complex regulatory networks that control gut health.

Claudins are essential constituents of tight junctions, which establish a physical barrier between intestinal epithelial cells (40). The inducible intestine-specific conditional deletion of *Cldn7* gene in mice (utilizing villin-CreERT2) resulted in heightened vulnerability to colitis, evidenced by higher weight loss and colon shortening (40). The mechanistic deletion of *Cldn7* compromised the intestinal mucus barrier and facilitated bacterial translocation to the intestinal epithelium (40). Analysis of microbiota by *16S* rRNA gene sequencing revealed that *Cldn7* deficiency decreased gut microbiota diversity and notably elevated the relative abundance of *E. coli* (40). Predicted functional analysis indicated enrichment of microbiota influencing infectious illnesses, immunological responses, and metabolic activities (40). The direct correlation between *Cldn7* knockout, impaired tight junctions, and distinct microbial changes illustrates that the structural integrity of tight junctions not only prevents systemic translocation but also actively influences the composition and diversity of the luminal microbiota by regulating their proximity to the epithelium. A weakened barrier selectively favors specific microbial populations, including *E. coli*.

The ablation of gut microRNAs (miRNAs) via intestinal epithelial cell-specific conditional deletion of the *Dicer1* gene in mice markedly modifies the gut microbiome (41). *Dicer1* is crucial for the processing of short non-coding RNAs, such as miRNAs, which modulate gene expression post-transcriptionally. The lack of *Dicer1* in intestinal epithelial cells affects butyrate-producing Firmicutes bacteria, which are recognized for preserving intestinal barrier integrity and mitigating inflammation, frequently resulting in a decline (41). Conversely, this deletion may result in an elevation of Proteobacteria, especially the *Escherichia*/*Shigella* genera, which are linked to toxin production and inflammatory activation (41). This discovery suggests that host gene expression, especially at the post-transcriptional stage through miRNAs, might significantly affect the makeup of gut microbiota. This indicates a complex regulatory feedback mechanism wherein host epithelial cells, via their miRNA profiles, can modulate the microbial environment, influencing the prevalence of favorable compared to potentially harmful species. The persistent finding that defects in host barriers, irrespective of the specific gene implicated, foster an environment favorable to the proliferation of pathobionts, which can subsequently intensify inflammation and disease, highlights that the capacity of the host to sustain a strong barrier is a fundamental selective pressure on the microbial community.

ACF7 (actin crosslinking family protein 7) belongs to the spectraplakin family of cytoskeletal crosslinking proteins, which controls cell shape by binding to actin and microtubules. Intestinal-specific *ACF7* deficiency markedly modifies gut microbiota composition, independent of dietary influences (42). This dysbiosis collaborates with a high-fat diet (HFD) to significantly impair intestinal barrier function. *ACF7* cKO animals demonstrate heightened intestinal permeability, epithelium apoptosis, and ultrastructural impairment of tight/gap junctions, which collectively trigger metabolic endotoxemia and ‘low-grade’ inflammation (36). Paradoxically, *ACF7* cKO diminishes nutrition absorption despite increased caloric intake. Under a high-fat diet (HFD), these abnormalities exacerbate, resulting in significant colonic inflammation, goblet cell hyperplasia, and metabolic alterations such as decreased cholesterol and triglycerides (42). Consequently, the absence of *ACF7* undermines gut barrier integrity and microbial equilibrium, intensifying inflammation and metabolic dysfunction generated by diet. *ACF7* is essential for preserving intestinal resilience in response to dietary stresses.

The *NHE3* (sodium-hydrogen exchanger 3) gene is essential for preserving the acid-base equilibrium of the body, and modulating salt and water reabsorption in the intestines and kidneys. Inducible deletion of intestinal-specific NHE3 in results in significant microbial dysbiosis, marked by elevated α-diversity and unique alterations in β-diversity (43). Significant alterations encompass an increase in pro-inflammatory Proteobacteria and pathobionts such as *Bacteroides thetaiotaomicron*, along with a decrease in beneficial Firmicutes, hence diminishing the Firmicutes/Bacteroidetes ratio, a characteristic indicative of dysbiosis (44). Species-level study identified increased abundance of several pathobionts (e.g., *Parabacteroides distasonis*) and depletion of gut commensal probiotics (e.g., *Roseburia hominis*). These alterations establish an alkaline, high-sodium luminal milieu resulting from compromised Na^+^/H^+^ exchange, hence promoting inflammatory bacteria (45). This dysbiosis clinically resembles alterations observed in IBD patients and elucidates the role of *NHE3* deficiency in predisposing individuals to IBD, as the modified microbiota promotes colitis via persistent inflammation and impaired barrier function (46).

### 2.2 Shaping microbial communities through immune and antimicrobial defenses

**Figure 2:**
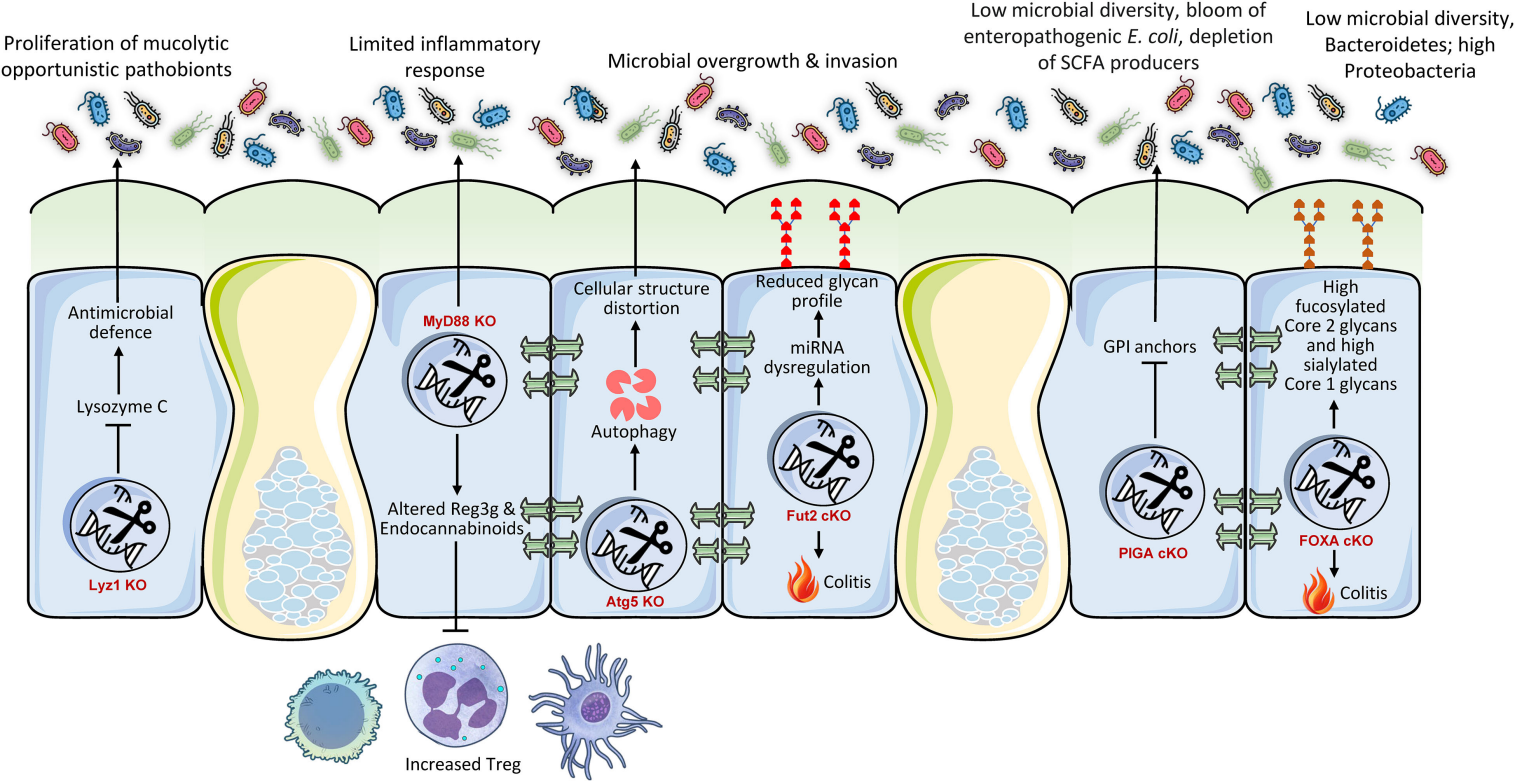
Conditional intestinal knockouts demonstrate that host genes regulating immunity, glycosylation, and cellular homeostasis are essential gatekeepers of microbial symbiosis. Mutations in immune effectors (Lyz1, MyD88), autophagy (Atg5), or glycan synthesis (Fut2, PIGA, FOXA) invariably result in dysbiosis, marked by a reduction of beneficial commensal bacteria and a proliferation of pro-inflammatory pathobionts. This alteration disturbs the gut ecological equilibrium, diminishes protective metabolites, and intensifies inflammation, resulting in phenotypes like aggravated colitis. These findings collectively illustrate that the host utilizes a multi-faceted defense mechanism to preserve a healthy microbiome and mitigate inflammation.

The gut immune system and its antibacterial properties are essential for differentiating commensals from pathogens and preserving the overall gut microbial equilibrium. cKO models have elucidated how particular host immunological elements within the colon directly affect the gut microbiota (**Figure 2**). For instance, Paneth cells, specialized secretory cells situated at the base of the intestinal crypts, synthesize antimicrobial peptides such as lysozyme C, which is encoded by the Lyz1 gene, to maintain microbial balance (37). Research employing a Paneth cell-specific cKO of Lyz1 in mice revealed that the lack of lysozyme C diminishes the immune response to bacterial molecular patterns in the gastrointestinal tract (47). This loss resulted in the proliferation of lysozyme-sensitive mucolytic bacteria, underscoring the pivotal role of Paneth cell-derived lysozyme in shaping the composition of mucolytic microbiota and preserving gut homeostasis (47). This illustrates that Paneth cells function as an essential ‘microbial filter’ in the intestine, actively influencing the local microbial ecology by selectively suppressing or facilitating the proliferation of specific bacterial species. The lack of this particular host defensive mechanism directly disrupts the microbial ecological equilibrium.

#### 2.2.1 Myeloid differentiation primary response 88

Myeloid differentiation primary response 88 (MyD88) serves as a pivotal adapter protein for the majority of Toll-like Receptors (TLRs), which are essential pathogen recognition receptors at the intersection of host-microorganism interactions (48). cKO of MyD88 in intestinal epithelial cells of mice offers partial protection against obesity, diabetes, and inflammation induced by calorie-rich diet (49). This protection was significantly linked to an enhanced metabolic profile and was transferable through gut microbiota transplantation to GF recipients, suggesting a direct involvement of the microbiota in facilitating these effects (49). The deletion resulted in elevated anti-inflammatory endocannabinoids, restored production of antimicrobial peptide (Reg3g), and an increase in intestinal regulatory T cells (49). The findings indicate that intestinal epithelial MyD88 functions as a sensor that modulates the gut microbiota composition according to nutritional status, subsequently affecting host energy metabolism and disease progression (49). This elucidates a complex mechanism wherein intestinal epithelial innate immunological signaling (via MyD88) functions as a ‘metabolic switch’ that converts dietary inputs into alterations in gut microbiota composition, thereby influencing systemic metabolic consequences. The immunological sensing of the intestinal environment directly influences the microbiota, hence affecting overall physiological conditions.

Autophagy is a vital biological mechanism essential for sustaining cellular homeostasis, particularly in the optimal operation of intestinal epithelial cells. A gut-specific cKO mouse model of Atg5 revealed that the interruption of autophagic flow in intestinal epithelial cells significantly modified the composition and reduced the diversity of the gut microbiota (50). Moreover, Paneth cells in Atg5-deficient mice exhibited morphological defects, establishing a direct connection between autophagy and the functionality of these essential antimicrobial-producing cells (50). This underscores that essential cellular mechanisms such as autophagy in intestinal epithelial cells, especially in specialized cells like Paneth cells, are crucial for preserving the structural and functional integrity necessary to sustain a healthy and diverse gut microbial community. Interruption of these fundamental biological mechanisms can produce cascading repercussions throughout the entire microbial ecosystem. The cumulative evidence from these immune-related knockouts identifies intestinal epithelial immune components as active ‘gatekeepers’ that, through their specific functions (antimicrobial secretion, signaling, cellular health), directly influence which microbial species can flourish and which are inhibited, thus preserving ecosystem equilibrium.

#### 2.2.2 Fucosyltransferase 2

Fucosyltransferase 2 (Fut2) is essential in the human gut for the synthesis of the H antigen, a precursor to ABO blood group antigens, on intestinal cell surfaces and blood (51). This synthesis is crucial for host-microbe interactions and may also provide protection against specific diseases. Deficiency of Fut2 specific to the intestinal epithelium markedly alters the structure and function of gut microbiota, resulting in adverse health consequences (52). This was justified by the fact that epithelial glycans are vital in modulating the gut microbiota by supplying bacterial ligands and nutrients, hence influencing the spatial structure of the gut microbiota (53). Fut2^ΔIEC^ Mice demonstrate significantly diminished microbial diversity and altered composition, defined by a reduction of advantageous families (e.g., Muribaculaceae, Ruminococcaceae) and an increase in pro-inflammatory gram-negative genera (*Escherichia*, *Bilophila*, *Enterorhabdus*, *Gordonibacter*). This dysbiosis induces a functional metabolic alteration, particularly augmenting choline/glycerophospholipid pathways and increasing the synthesis of lysophosphatidylcholine (LPC) through heightened microbial phospholipase A activity. Thus, Fut2 deficiency intensifies colitis severity during dextran sodium sulfate challenge (54). It exacerbates inflammation by increasing pro-inflammatory cytokines (TNF-α, IL-1β, IL-6; p < 0.05) and macrophage infiltration (threefold increase, p = 0.001), while compromising epithelial barrier integrity through decreased tight junction proteins and reduced mucus/goblet cells. The loss of Fut2 collectively alters the gut microbiota, promoting a pro-inflammatory, barrier-disrupting condition that increases vulnerability to IBD (55).

#### 2.2.3 Glycosylphosphatidylinositol-anchored proteins

Glycosylphosphatidylinositol-anchored proteins (GPI-APs) have a role in various gut-associated immunological processes, such as microbial surveillance, defense, and epithelial cell polarity (56). The PIGA (phosphatidylinositol glycan anchor biosynthesis, class A) gene encodes the protein phosphatidylinositol glycan class A, which is crucial for the production of GPI anchors (57). Intestinal epithelium-specific Piga deletion in mice markedly modified gut microbiota composition and function, adversely affecting health (58). Specifically KO mice demonstrated diminished microbial diversity and a significant proliferation of enteropathogenic *E. coli*, generally associated with endotoxemia and inflammation (4). Simultaneously, SCFA-producing bacteria (e.g., *Faecalibacterium prausnitzii*, *Roseburia* spp.) were diminished. The microbiota in knockout mice exhibited reduced potential for vitamin biosynthesis, while demonstrating increased functions pertaining to pathogen survival under inflammatory conditions. Therefore, a lack of PIGA indicates increased vulnerability to persistent gastrointestinal inflammation and metabolic disorders as seen in the case of IBD patients (59).

FOXO transcription factors govern numerous cellular processes in the gut, encompassing cell destiny, immunological responses, and cellular quality control, all of which are vital for optimal gut function and homeostasis. Ablation of intestinal FOXA1/2 transcription factors in mice impaired epithelial glycosylation, notably diminishing fucosylated Core 2 glycans and augmenting sialylated Core 1 glycans in mucus (60). The modified glycan profile resulted in significant dysbiosis, marked by diminished microbial diversity, a reduction in beneficial *Bacteroidetes*, and an increase in Proteobacteria (e.g., *Sutterella*) that contributes to mucosal inflammation (61). As a result, FOXA-deficient animals exhibited spontaneous colitis, characterized by weight loss, increased fecal lipocalin-2, crypt hyperplasia, and immunological infiltration. Antibiotic therapy mitigated colitis, substantiating the role of the microbiota. Importantly, fecal transplants from knockout to wild-type mice did not maintain dysbiosis, highlighting that host-derived glycans predominantly influence a healthy microbiota. Consequently, FOXA-dependent glycosylation is crucial for microbiome symbiosis, and its impairment directly induces inflammation and colitis.

### 2.3 Regulating microbiota via nutrient metabolism and signaling pathways

**Figure 3:**
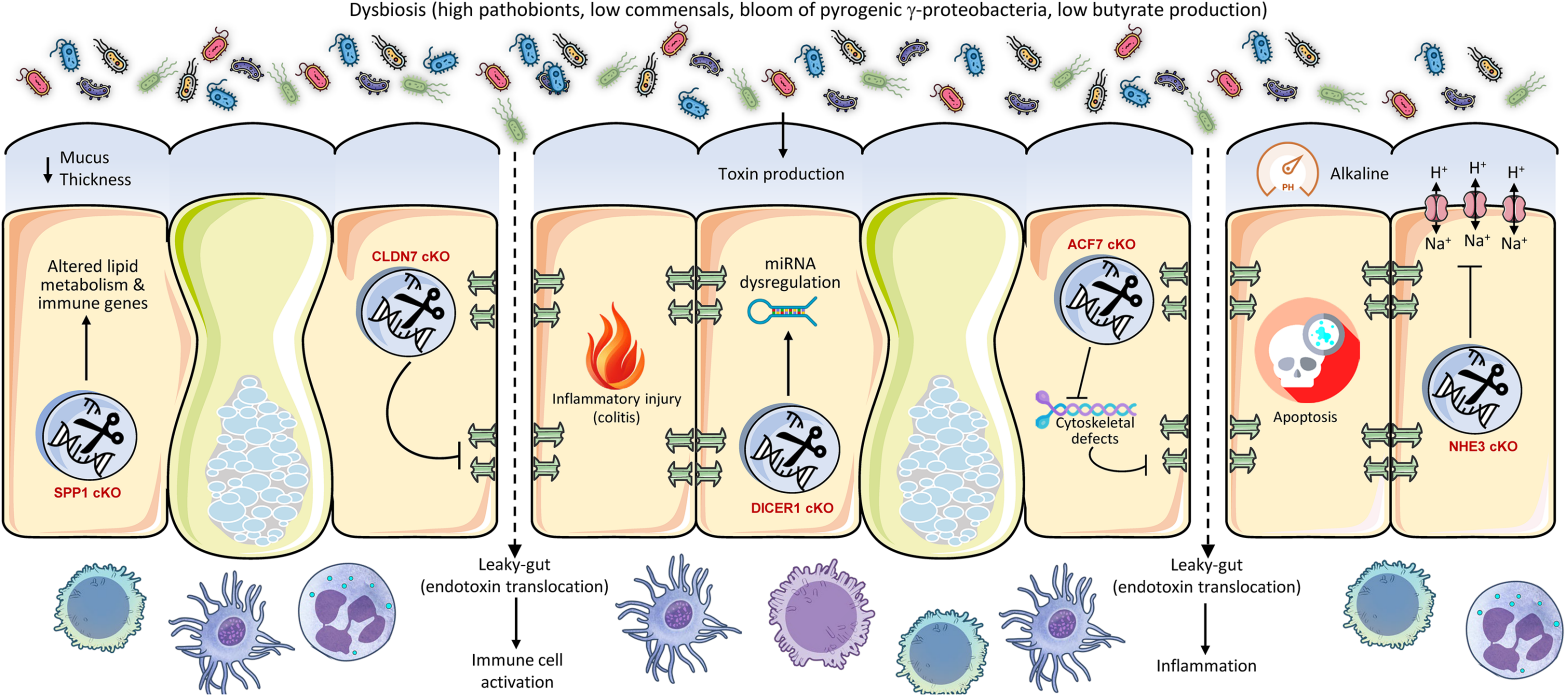
Conditional intestinal knockouts of metabolic regulators demonstrate their pivotal role as gatekeepers of host-microbe symbiosis. Genes controlling bile acid sensing (FXR), glucose transport (GLUT2), glycolysis (HK2), mitochondrial function (HSP60), and vitamin signaling (VDR) profoundly shape the gut luminal environment. Their deletion alters nutrient availability and epithelial health, leading to dysbiosis characterized by a loss of beneficial butyrate-producers and an expansion of opportunist pathobionts like E. coli. This metabolic disruption increases susceptibility to colitis and metabolic disorders, underscoring that host metabolism actively maintains a healthy microbiota to prevent inflammation and disease.

In addition to its barrier and immunological roles, the intestinal epithelium plays a crucial role in nutrition absorption and metabolic signaling, significantly impacting the gut microbiota (**Figure 3**). For instance, bile acids, produced from cholesterol in the liver, are essential signaling molecules that also influence gut microbiota composition and metabolic functions. The intestinal epithelial receptors, Farnesoid X Receptor (FXR) and Takeda G protein-coupled receptor 5 (TGR5), are integral to these activities, affecting intestinal absorption, energy metabolism, and the regulation of gut microbiota (62). Mice with intestinal-specific FXR deficiency exhibited resistance to obesity, insulin resistance, and non-alcoholic fatty liver disease generated by a high-fat diet (63). The protective effect was associated with modifications in the gut microbiota, characterized by reduced levels of the genus *Lactobacillus* and diminished bile salt hydrolase (BSH) activity, leading to elevated concentrations of tauro-β-muricholic acid (T-β-MCA), a powerful FXR antagonist (63). This illustrates that intestinal FXR significantly impacts host metabolic health by modulating bile acid metabolism and the resulting microbiota composition (63). The gut microbiota is essential for sustaining the postprandial glucagon-like peptide-1 (GLP-1) response, especially in the ileum (64). Bile acid-TGR5 signaling is implicated in this process, with certain bile acids (ωMCA and HCA) promoting GLP-1 release through TGR5 (64). Fecal microbiota transplantation or supplementation with these bile acids reinstated the postprandial GLP-1 response, highlighting the significance of the microbiome in TGR5-mediated metabolic regulation (64). The modulation of bile acid receptors in the intestine results in alterations to microbiota and significant metabolic consequences, including modified obesity susceptibility and GLP-1 secretion. The modification of bile acids by the microbiota underscores a complex ‘gut-liver-microbiota’ axis, wherein host intestinal receptors detect bile acids that are influenced by the microbiota. This sensing then affects host metabolism, which in turn regulates the microbiota. The capacity of the host to detect and react to these microbial-altered metabolites constitutes a crucial regulatory juncture.

The intestinal glucose transporter 2 (GLUT2) is involved in glucose absorption and maintaining gut homeostasis (65). Intestinal GLUT2 knockdown in mice was observed to maintain gut integrity, diminish infection susceptibility, and markedly alter the makeup of gut microbiota (65). This also resulted in a decrease in systemic inflammation. This illustrates that host metabolic processes, particularly food transport, encompass not just energy acquisition but also the establishment of the metabolic niche for gut bacteria. Modifying nutrient availability via host transporters might directly favor or disfavor particular microbial populations, hence influencing the whole ecosystem. Hexokinase 2 (HK2) is significantly expressed in the intestinal epithelium and is crucial to glycolysis (66). Researchers discovered that animals with epithelial HK2 deletion, achieved by the Cre-loxP system, had reduced susceptibility to acute colitis (67), and that the ablation of epithelial HK2 inhibits cellular growth and disrupts mitochondrial activity in neoplastic epithelial cells, hence providing protection against intestinal injury (68). A metabolite generated from probiotic microbes, butyrate, was demonstrated to inhibit HK2 expression and safeguard wild-type mice from colitis (69). This suggests that intestinal butyrate, an essential SCFA generated by gut microbiota, enhances intestinal hemostasis by inhibiting epithelial HK2, therefore reducing intestinal inflammation. This illustrates an intriguing reciprocal regulatory loop in which a host metabolic enzyme (HK2) affects intestinal health, while its activity is subsequently regulated by a crucial microbiota-derived metabolite (butyrate). Thus, host metabolic pathways can be modulated by microbial signals, underscoring a profound integration of host and microbial metabolism in sustaining gut homeostasis (3). The intestine functions not merely as a ‘host’ for bacteria but as an active ‘provider’ that nurtures various microbial populations by regulating the availability of nutrients and signaling molecules. The metabolic condition of the host directly affects the microbial population, which subsequently generates compounds that feedback to impact the metabolism of the host. This bidirectional metabolic regulation is essential for health.

Recent study show that the gut bacterial communities adjust to changes in epithelial metabolism, creating a dysbiotic adaptation of the microbiota. Specifically, mitochondrial dysfunction at the intestinal mucosa, resulting from Hsp60 deletion, initiated self-resolving tissue injury in mice; however, this injury advanced to a severe IBD-like condition when coupled with suppression of IL-10 or AhR, which are crucial regulators of gut homeostasis (70). This injury was critically dependent on microbiota, as GF animals or antibiotic treatment inhibited distal colon damage. Mitochondrial dysfunction induced dysbiosis marked by the proliferation of metabolically adaptable *Bacteroides* spp., especially *B. caecimuris*, which alone reproduced the damage phenotype following mono-colonization. This dysbiosis impaired mucus synthesis and epithelium renewal, exacerbating inflammation, similar to IBD (71). Transcriptional profiling identified a metabolic injury signature (e.g., IDO1, NOS2, DUOX2) that differentiated inflamed from non-inflamed tissue in Crohn’s disease patients, associating mitochondrial dysfunction and Bacteroides dysbiosis with IBD progression via disrupted host-microbe metabolic interactions.

Finally, vitamin D in the intestine, through its active form binding to the vitamin D receptor (VDR), performs various functions by augmenting calcium and phosphate absorption, fortifying epithelial barrier integrity via the upregulation of tight junction proteins and antimicrobial peptides, modulating innate and adaptive immune responses to maintain homeostasis, and fostering a balanced microbiota to safeguard against inflammation, infection, and intestinal disorders (72, 73). The intestine-specific knockdown of the VDR markedly altered the makeup of the gut microbiome, resulting in dysbiosis (74). This was accompanied by a significant reduction in beneficial butyrate-producing bacteria, essential for gut health and anti-inflammatory benefits. Particular adverse changes included a rise in *E. coli* prevalence and a decline in essential taxa, including *Prevotella dentalis*, *Akkermansia muciniphila*, and *Parabacteroides* spp., including *P. distasonis* and P. sp. CT06. The increase in *E. coli* was functionally associated with modifications in host carbohydrate metabolism, especially maltose processing. This VDR-mediated dysbiosis collectively leads to adverse effects, such as compromised intestinal autophagy and heightened vulnerability to illnesses like colitis, highlighting the essential function of VDR in preserving microbial equilibrium.

## 3 Unifying mechanisms and emerging understandings

The collective findings from intestine-specific cKO studies reveal unifying themes in how the host orchestrates and regulates its gut microbiota. These mechanisms extend beyond simple physical containment to encompass sophisticated immune, metabolic, and cellular processes.

### 3.1 Host genetic control of microbiota through epithelial barrier function

The intestinal barrier, consisting of the mucus layer, epithelial cells, and tight junctions, functions as the essential interface regulating host-microbiota interactions (36), with host genetics significantly influencing its integrity and function (75), as demonstrated by intestinal-specific cKO studies. The targeted deletion of the Spp1 (osteopontin) gene undermines the integrity of the mucus layer, markedly decreasing mucin staining and colonic mucus thickness (39). This reduction compromises the physical separation of luminal bacteria, allowing direct microbial-epithelial contact, which incites inflammation and modifies microbial diversity, as demonstrated by metagenomic alterations in bacterial pathways associated with lipid metabolism and inflammation (76). Likewise, Cldn7 cKO directly undermines tight junction integrity (40), elevating intestinal permeability and facilitating bacterial translocation, which preferentially promotes the proliferation of pathobionts such as *E. coli* and diminishes overall microbial alpha diversity, as functional predictions suggest an enrichment for pathways associated with inflammatory and infectious diseases (77, 78). The disruption of host post-transcriptional regulation through Dicer1 cKO, which eliminates epithelial microRNA biogenesis, results in the depletion of beneficial butyrate-producing Firmicutes, essential for barrier reinforcement via SCFAs (79), while fostering the proliferation of Proteobacteria such as *Escherichia*/*Shigella* (61), thereby illustrating how host RNA-mediated gene expression influences a microbial environment conducive to dysbiosis. Cytoskeletal integrity is essential, as ACF7 cKO disrupts junctional ultrastructure (tight/gap junctions) and elevates epithelial apoptosis and permeability (42). This barrier dysfunction synergizes with dietary stressors such as a high-fat diet to induce significant dysbiosis, metabolic endotoxemia, and inflammation (80), while paradoxically hindering nutrient absorption despite heightened caloric intake. Ion transport is equally vital, as the cKO of NHE3 (Na^+^/H^+^ exchanger) disrupts luminal Na^+^/H^+^ exchange (43), resulting in an alkaline, sodium-rich luminal environment that fosters pro-inflammatory pathobionts while depleting commensal probiotics (81), thus reducing the Firmicutes/Bacteroidetes ratio and reflecting the dysbiotic profile characteristic of IBD (82). These genetic models collectively clarify three fundamental processes via which host genes regulate the microbiota across the barrier: (i) Physical barrier failure, characterized by defects in mucus (Spp1) or junctions (Cldn7, ACF7) that facilitate pathobiont adherence and translocation; (ii) Metabolic niche alteration, wherein host-induced modifications in luminal microenvironment (e.g., NHE3-mediated alkalinization, ACF7/Spp1-influenced metabolism) or nutrient availability favors specific bacterial taxa; and (iii) Immune-microbe feedback, in which initial barrier defects incite inflammation (Spp1, Cldn7), further altering microbial composition towards a pro-inflammatory condition. This genetic regulation exerts a fundamental selective pressure on the microbial population (83), with barrier deficiencies continually promoting pathobiont dominance (particularly Proteobacteria such as *E. coli*) and diminishing variety, ultimately predisposing individuals to disease such as IBD and metabolic syndrome. Thus, treatment approaches aimed at these gene-barrier-microbiota interactions, such as augmenting mucin synthesis, reestablishing ionic equilibrium, or administering microbial metabolites like butyrate, show potential for reinstating microbial homeostasis.

### 3.2 Immune system components as determinants of microbial homeostasis

The gut immune system demonstrates hierarchical genetic regulation of microbiota composition via epithelial antimicrobial defense, immunological signaling, and glycan-mediated niche modification, as substantiated by intestinal-specific cKO animal models. The targeted deletion of Lyz1 in Paneth cells eliminates lysozyme C secretion (47), undermining antibacterial responses against mucolytic bacteria and disrupting microbial balance by allowing the proliferation of lysozyme-sensitive taxa (3). Concurrently, Atg5 cKO, which disrupts autophagy, causes morphological defects in Paneth cells and hinders antimicrobial peptide storage, collectively diminishing microbiota diversity and facilitating dysbiosis (50). Immune signaling pathways function as microbial sensors; the deletion of MyD88 in intestinal epithelial cells reconfigures the microbiota by increasing anti-inflammatory endocannabinoids and regenerating islet-derived protein 3 gamma (Reg3γ), thereby providing protection against diet-induced metabolic disease, effects that are transferable through fecal transplant (49). This underscores how innate immune adaptors convert nutritional signals into microbiota-mediated metabolic consequences. Conversely, errors in glycosylation have significant effects, such as the Fut2 cKO reduces epithelial fucosylation (Tang et al., 2021), which is essential for mucosal protection from pathogens (84, 85). This undermining colonization resistance by depleting advantageous Muribaculaceae and Ruminococcaceae, while promoting pro-inflammatory Gram-negative genera (e.g., *Escherichia, Bilophila*). This alteration elevates microbial phospholipase A activity, increases lysophosphatidylcholine (LPC), and intensifies colitis severity during chemical exposure (86). Likewise, the ablation of FOXA1/2 disrupts core fucosylation and increases sialylation (60). This results in the bloom of *Sutterella* (Proteobacteria) at the expense of Bacteroidetes and inducing microbiota-dependent spontaneous colitis (resolved by antibiotics) that is not transferable to wild-type hosts, highlighting that host-derived glycans uniquely govern microbial symbiosis (87). Moreover, Piga cKO impairs the functionality of glycosylphosphatidylinositol (GPI)-anchored proteins, leading to an increase in pathogenic *E. coli* strains (e.g., O157:H7) and a reduction in SCFA producers (*Faecalibacterium*, *Roseburia*), while also decreasing microbial vitamin synthesis and enhancing virulence pathways (58), thus establishing an inflammatory environment reminiscent of IBD (88, 89).

These genomic alterations converge on three fundamental mechanisms: (i) antimicrobial function depletion, characterized by impaired Paneth cell function (Lyz1, Atg5) that allows for pathobiont invasion (90). Paneth cells, situated in the intestinal crypts, release antimicrobial peptides such as α-defensins that modulate microbial populations (3). Impairment of Paneth cells, resulting from genetic mutations, inflammation, or environmental factors, compromises this antimicrobial defense, diminishing microbial regulation. This results in the proliferation of pathogenic bacteria and the depletion of helpful commensals, causing an imbalance in gut microbiota (90). Dysbiosis exacerbates intestinal inflammation and conditions, including IBD. Mutations in the ATG16L1 gene compromise Paneth cell functionality and are associated with dysbiosis in Crohn’s disease (91). Consequently, Paneth cell failure is crucial in maintaining gut microbial equilibrium. (ii) Signal dysregulation, in which disrupted immune sensing (MyD88) disrupts diet-microbiota interactions. MyD88 and TLR-dependent immune signaling are essential for coordinating the complex host-microbiota interaction and for preserving intestinal homeostasis (48). It allows the host to distinguish between gut commensals and pathogens, initiate suitable immune responses against invasive microbes, and foster tolerance towards the commensal microbiota (92). MyD88-dependent signaling affects gut microbiota composition, maintains intestinal barrier integrity, and regulates the growth and function of immune cells, such as regulatory T cells and the synthesis of antimicrobial peptides (93, 94). Dysregulation of this system can disturb the fragile equilibrium, resulting in heightened vulnerability to infections and chronic inflammatory disorders such as IBD. (iii) Glycan Niche Collapse, where abnormal fucosylation (Fut2, FOXA) or loss of GPI-anchor (Piga) removes adhesion sites for commensals, facilitating pathobiont proliferation (*Escherichia*, *Sutterella*) and diminishing anti-inflammatory taxa. As a result, host immune genes establish ‘colonization resistance’ through peptide secretion, glycan-lectin interactions, and immunometabolic signaling; their disruption leads to pathobiont-dominated dysbiosis, diminishes SCFA production, and heightens inflammation. Therapeutic approaches aimed at these axes, such as fucosylated glycan mimetics to reinstate Fut2-mediated symbiosis or microbiota transplantation to rectify MyD88-associated communities, show potential for IBD and metabolic disorders, highlighting that intestinal immune genetics serve as principal regulators of microbial community.

### 3.3 Metabolic pathways and signaling molecules as key mediators

The intestinal epithelium genetically regulates gut microbiota composition via nutrient sensing, transport, and metabolic signaling pathways, creating a bidirectional regulatory axis that affects microbial ecology and disease risk. Intestinal-specific ablation of the bile acid receptor FXR imparts resistance to diet-induced obesity through modifications in bile acid metabolism (63). FXR deficiency increases tauro-β-muricholic acid (T-β-MCA), which inhibits bile salt hydrolase (BSH)-producing *Lactobacillus*, thereby diminishing bile acid deconjugation and enhancing T-β-MCA accumulation in a self-perpetuating cycle that influences host metabolism. Simultaneously, the activation of the TGR5 receptor by microbially altered bile acids (e.g., ωMCA, HCA) promotes the secretion of glucagon-like peptide-1 (GLP-1) (3), a mechanism reliant on gut microbiota, as fecal transplantation reinstates postprandial GLP-1 responses, exemplifying a ‘gut-liver-microbiota’ axis wherein host receptors identify microbial metabolites to modulate systemic energy homeostasis (95). On the other hand, nutrient transporters can delineate microbial niches. GLUT2 knockdown diminishes luminal glucose efflux, modifies microbiota composition, enhances barrier integrity, and decreases inflammation (65). This illustrates that host transporters shape microbial environments by regulating substrate availability (96, 97). Likewise, a deficit in the riboflavin transporter RFVT-3 hinders vitamin absorption, leading to oxidative stress and dysbiosis marked by reduced *Bifidobacterium*, which is reversible solely by riboflavin supplementation (98). Metabolic enzymes participate in reciprocal interactions with metabolites derived from microbiota; HK2, a regulator of glycolysis, is inhibited by butyrate, produced by commensals such as *Faecalibacterium*, and the deletion of epithelial HK2 confers protection against colitis (67), elucidating a feedback loop where microbial metabolites attenuate host glycolysis to mitigate inflammation. Mitochondrial failure, such as by Hsp60 deletion, induces dysbiosis characterized by the prevalence of metabolically flexible *Bacteroides caecimuris* (70), which degrades mucus barriers and hinders epithelial regeneration (99), hence intensifying inflammation in a microbiota-dependent manner. Disease-specific signatures arise from these interactions. In IBD, markers of mitochondrial dysfunction (e.g., IDO1, DUOX2) correlate with the expansion of Bacteroides, whereas vitamin D receptor (VDR) deficiency modifies bile acid metabolism and reduces SCFA producers (e.g., *Clostridium* spp.), thereby heightening susceptibility to colitis (74). These genetic understanding of microbial regulation converge on a framework where host metabolic genes, through bile acid signaling, nutrient flow regulation, enzymatic functions, and organellar activities, establish luminal metabolic niches that favor either commensals or pathobionts. Therapeutic approaches aimed at these axes show potential, highlighting that human metabolism serves as a principal architect of microbial communities via nutrient-driven selection pressures.

## 4 Future directions and therapeutic potential

Intestine-specific cKO studies have conclusively demonstrated the host-mediated active genetic modulation of the gut microbiota via barrier integrity, immunological responses, and metabolic signaling, identifying crucial targets for therapeutic intervention. Future research should focus on enhancing mechanistic comprehension by investigating unexamined genes within these pathways (e.g., particular tight junction proteins, antimicrobial peptides, nutrient sensors) and utilizing longitudinal, multi-omics techniques to elucidate the temporal dynamics of dysbiosis induction and microbial resilience in response to acute versus chronic genetic perturbation. This line of studies could be directly applicable to practical therapeutic practices. One strategy entails fortifying the impaired intestinal barrier, as demonstrated in animals such as Spp1 (mucus depletion) or Cldn7 (tight junction failure) cKO. This can be accomplished using mucin secretagogues (100), probiotics that augment goblet cell activity (101), or agents that maintain epithelial junctions to inhibit pathobiont transfer. Another technique could be to mitigate the reduction of advantageous metabolites, specifically butyrate, noted in Dicer1, VDR, or PIGA cKO models. This can be resolved by the precise administration of butyrate prodrugs (102) or the application of engineered butyrate-producing probiotics (103), which may additionally target downstream inflammatory pathways, including HK2 inhibition. A third treatment approach could focus on reinstating the glycan niche compromised by Fut2 or FOXA deficiency. This can be achieved through the utilization of fucosylated prebiotics (104) or glycan-mimetic drugs (105) to specifically support commensals (e.g., *Ruminococcaceae*) and impede the adherence of pro-inflammatory genera such as *Escherichia*. Ultimately, regulating essential host receptors, like bile acid sensors FXR and TGR5, dysregulated in cKO studies can aid in correcting microbiota-related metabolic disorders (106). This can be accomplished through the utilization of tissue-specific agonists or antagonists to mitigate systemic negative effects (107).

The capacity of intestine-specific cKO to accurately impair host gene function provides a framework for innovative treatment approaches. Future therapies should prioritize the modulation of specific host pathways within the intestine, rather than exclusively targeting the microbiota (e.g., probiotics, fecal microbiota transplantation), to foster a more conducive environment for the gut commensals or to inhibit the pathobionts. Pharmacological agonists or antagonists targeting host receptors such as FXR or TGR5 could be formulated to indirectly modify the microbiota and affect metabolic health (63). Likewise, approaches to augment Paneth cell functionality or mucus synthesis may be investigated to reestablish microbial balance. This precision medicine strategy, guided by cKO investigations, signifies a fundamental transformation towards host-centric microbiota modulation. This advocates for a host-centric methodology in microbiota therapeutics, wherein interventions are crafted to enhance the intestinal milieu (e.g., barrier integrity, immune signaling, metabolic pathways) of the host to inherently promote a healthy microbiome, rather than merely introducing or eliminating microbes. This may result in more consistent and individualized therapy results.

Nonetheless, the translation of these promising pathways encounters considerable obstacles, including the necessity of ensuring human relevance beyond murine models via human intestinal organoids or ‘humanized’ microbiota transplants, attaining enhanced cell-type specificity (e.g., Paneth cell-exclusive knockouts) to elucidate mechanisms, incorporating the roles of fungi, viruses, and phages within the comprehensive microbial community, and devising more precise microbiome-modulating therapies, such as engineered microbial consortia or targeted phage therapy against specific pathobionts (e.g., *E. coli* blooms), to supplant the rudimentary approach of fecal microbiota transplantation (FMT). Consequently, the future of cKO-driven research resides in utilizing these profound mechanistic insights to devise spatially and temporally regulated interventions that accurately target host pathways influencing microbial ecology, ultimately reinstating symbiosis to address conditions such as IBD, metabolic syndrome, and opportunistic infections without compromising systemic homeostasis, advancing from mere observation to the active engineering of a healthy host-microbe interface through sustained symbiosis.

## 5 Conclusion

Intestine-specific cKO studies have been essential in elucidating the intricate and frequently neglected function of the host in influencing the gut microbiota. Intestine-specific cKO mice provide substantial benefits over whole-body KOs for investigating host impacts on gut microbiota by eliminating confounding systemic influences. They mitigate mortality or developmental anomalies resulting from important gene deletion in other regions, eradicate secondary effects from remote organs (e.g., immune system, liver), and accurately delineate the function of the target gene inside intestinal cells (e.g., epithelium, immune cells). This facilitates direct examination of local intestinal pathways influencing the microbiota. Although only a limited number of studies have been undertaken to date, intestinal cKO studies have illustrated how host variables associated with barrier integrity, immunological defense, and metabolic signaling directly affect microbial diversity, composition, and function. Essential discoveries underscore the pivotal functions of host tissue-specific genes in preserving microbial balance and mucosal immunometabolic homeostasis. These results transcend correlation observations, revealing causal relationships between host genetics and the gut microbiome. Ongoing studies into these complex connections are expected to facilitate the development of novel diagnostic instruments and host-targeted treatment approaches designed to modify the gut microbiota for enhanced human health.
